# Applicability of a Novel Attunement Instrument and Its Relationship to Parental Sensitivity in Infants With and Without Visual Impairments

**DOI:** 10.3389/fpsyg.2022.872114

**Published:** 2022-05-03

**Authors:** Victorita Stefania Vacaru, Andrea Urqueta Alfaro, Nadia Hoffman, Walter Wittich, Micky Stern, Heather J. Zar, Dan J. Stein, Paula Sophia Sterkenburg

**Affiliations:** ^1^Donders Centre for Cognitive Neuroimaging, Donders Institute for Brain, Cognition and Behaviour, Radboud University, Nijmegen, Netherlands; ^2^Vrije Universiteit, Amsterdam, Netherlands; ^3^Nazareth and Louis-Braille Institute, Integrated Health and Social Services Centres (CISSS), Longueuil, QC, Canada; ^4^School of Optometry, Université de Montréal, Montreal, QC, Canada; ^5^Department of Psychiatry and Mental Health, Faculty of Health Sciences, University of Cape Town, Cape Town, South Africa; ^6^Institut Nazareth et Louis-Braille du CISSS de la Montérégie-Centre, Longueuil, QC, Canada; ^7^MRC Unit on Child and Adolescent Health, Department of Paediatrics and Child Health, University of Cape Town, Cape Town, South Africa; ^8^Department of Paediatrics & Child Health, Red Cross War Memorial Children’s Hospital, Cape Town, South Africa; ^9^Risk and Resilience in Mental Disorders Research Unit, South African Medical Research Council, Cape Town, South Africa; ^10^Bartiméus, Zeist, Netherlands

**Keywords:** visual impairment, attunement, parental sensitivity, parent–infant interaction, instrument validation

## Abstract

This study investigated the applicability of a novel instrument to assess parent–child attunement in free play interactions, in dyads with an infant with and without visual impairments (VI). We here report the findings on the reliability and applicability of the newly developed *Attune & Stimulate Mother–Infant 56-items Instrument (A&S M-I)* in two separate samples: one with infants with VI (*N* = 20) and one with typically sighted infants (*N* = 24). In addition, we assessed the contribution of parental sensitivity to attunement in dyadic interactions. The *A&S M-I* is an observational comprehensive instrument of behaviors that captures different body parts and their motility (i.e., finger movements, arm waving, and foot kicking), and different senses (i.e., audio, tactile, and visual). The appropriate responding of a parent to the child’s signal (i.e., matching and containing) reflects the ability to attune in the dyad as well as parent’s ability to stimulate the child to become engaged in the contact or activity. Consistency assessments revealed good reliability for maternal and infant behaviors, acceptable internal consistency and good test–retest reliability. Furthermore, both samples scored significantly above chance level on attunement, suggesting that the instrument captures parent–infant behavioral coordination, and VI was not related to parent–infant attunement. Lastly, a relation between parental sensitivity and attunement was found only in the TS sample. Altogether, these findings provide promising initial evidence of the applicability of the *A&S M-I* instrument for assessing dyadic attunement across different populations and ages. Having assessed the applicability of this observational instrument, future work should corroborate these findings in larger samples.

## Attunement in Early Social Interactions

The foundation of infant development lies in the early caregiving environment and particularly in parent–infant face-to-face interactions (Ramey and Sackett, 2000; Beebe et al., 2016). The quality of such interactions, largely determined by how sensitive parents are and the capacity to coordinate their behavior to the infant’s needs, henceforth attunement, contributes to a myriad of developmental outcomes that span from physical to social cognitive and emotional areas (van der Voort et al., 2014; Vacaru et al., 2019, 2020, 2022). Attunement entails reciprocal behaviors of the mother and her infant, comprising maternal sensitive behaviors such as attention to infant’s cues, correct and timely interpretation and appropriate responsiveness (Bornstein and Manian, 2013; Lev-Enacab et al., 2022). It has been shown that mother’s attunement fosters infant’s developing abilities to share their inner states, and to later achieve intersubjectivity and better communication skills (Stern et al., 1985; Užgiris, 1991; Nicely et al., 1999), and joint engagement (Legerstee et al., 2007; Rollins and Greenwald, 2013). More importantly, since the early days of life, infants have an active role in forging the relationships with their parents, and although parents hold greater control and flexibility in regulating the interaction, they both contribute different yet intertwined elements to it (Provenzi et al., 2018; Puura et al., 2019). In typically sighted (TS) dyads, early parent–infant communication revolves around the face and gaze, and is characterized by synchrony and facial affective matching (Lester et al., 1985; Beebe and Steele, 2013; Moore et al., 2016). Moreover, maternal contingent responding to their infants was found to be related to infant’s gaze and led to positive affect manifested through facial expressions (Symons and Moran, 1987). These interactions seemingly rely mainly on infants’ visual capacities to perceive and respond appropriately to the parents, but an important question to raise is how do parents and infants with a visual impairment (VI), who cannot rely on visual input coordinate their behaviors and reach attunement.

## Visual Impairments

Visual impairments during early development may affect the quality of the parent–infant relationship (Howe, 2006; Sterkenburg et al., 2022): infants with VI may not be able to capture the range of their parent’s visual cues, whereas TS parents may miss or misinterpret their infant’s cues (Nagayoshi et al., 2017; van den Broek et al., 2017). Infants with VI may communicate differently, by using a unique set of signals, such as tactile strategies (Chen and Downing, 2006), which parents may not be aware of and hence may not perceive these as meaningful. A recent study indicated that the lower infant’s visual acuity (VA), the lower was their ability to share attention with their parent (Urqueta Alfaro et al., 2021). Consequently, parents may fail to respond to infant’s signals and stimulate the infant (Platje et al., 2018) or may become directive and intrusive (for a systematic review see: Grumi et al., 2021). For example, infants who are blind or have a severe VI may not make eye contact with their parents nor engage in reciprocal imitation games of facial expressions, interactions that are documented in TS infants during the first months of life (Beebe et al., 2010; Markova, 2018; Vacaru et al., 2022). Instead, infants with VI may react to their parent’s approach by making lips or tongue movements, as well as by waving legs and arms (Preisler, 1991). It is important to note that the impact of VI in child development varies depending on factors such as the severity of the VI (ranging from no light perception to low vision), and the parents’ ability to adapt their child-rearing practices to the unique needs of infants with VI (Warren et al., 1997; Lueck, 2008). To alleviate the strain of VI on the parent–infant relation and to mitigate the potential profound detrimental effects on infants’ subsequent development, it is crucial to identify the central communicative signals in the parent–infant interaction in the presence of VI. Identifying these signals hold important implications for supporting early intervention programs (Overbeek et al., 2015) to promote parent–infant attunement and psychological wellbeing (Kúld et al., 2020).

## Assessing Attunement in Mother–Infant Interactions

To assess dyadic attunement in mother–infant interactions, it is crucial to identify the behaviors that are typically displayed by infants. These behaviors can vary according to the infant’s temperament and/or context and it has been shown that parents can attune to their infant’s spontaneous movements from an early age (Lev-Enacab et al., 2022). Parental attunement to their infants’ behaviors is marked by sensitive responding to the infant’s cues (i.e., mirroring and emotional availability). The assessment of dyadic attunement is of great importance for clinical work with populations in which verbal communication or intellectual abilities may be limited, and interventions aimed at improving the relationship are needed. Particularly, a recent systematic review of children with VI concluded that research on mother–child interaction in this population is scarce, but the extant evidence underscores the importance of addressing exchange challenges between mothers and their child with a VI (Grumi et al., 2021; Provenzi et al., 2021). Indeed, in order to develop appropriate interventions, concrete behaviors need to be identified in the interaction and target those behavioral domains that are disrupted. To our knowledge, coding schemes for mother–child interaction quality have mostly focused on mothers’ behaviors, such as maternal sensitive responses to the infant or the child (for a systematic review see: Mesman and Emmen, 2013), here instead we want to focus on assessing the degree of overlap of behaviors of mothers and their infants. This is in line with the burgeoning literature underscoring the importance of dyadic biobehavioral synchrony for later socioemotional development (e.g., Feldman, 2012). Moreover, this instrument provides concrete examples of behaviors to observe, rather than broader categories that may be freer to objective interpretation.

## Attune & Stimulate Mother–Infant

Originally, an instrument named Attune & Stimulate was developed for caregivers of individuals with intellectual disabilities, in which several video recordings of client–therapist interactions were observed to identify concrete behaviors that were challenging in the exchange between a caregiver and an individual with disabilities wherein verbal communication was limited, and therefore, the coding relied mostly on behavioral/postural aspects (Doodeman et al., 2019). Observed behaviors were categorized along several dimensions, namely vocalizations-sounds, mouth movements, eyes-gaze, actions, body posture, upper or lower body, hands, arms, feet, and head. Using this instrument, helped to provide feedback to the caregivers about how they could attune more to the behaviors shown by the client. For instance, if the client would vocalize, the caregiver was given the tip to also vocalize and mirror the observed behaviors. Having as a starting point this observational framework, an adapted instrument named *Attune & Stimulate Mother–Infant (A&S M-I)* was developed from observations of mother–infant interactions. The coding involved both partners in the dyad and coders marked C if the behavior occurred in the child and P for the parent whenever a certain behavior occurred for one of them. If C and P were marked for one behavior, this was counted as an attunement instance (Doodeman et al., 2019, 2022). While there is initial indication based on this qualitative investigation, it is not known yet how this instrument can capture meaningful mother–child interaction behaviors across several populations and ages, and whether attunement as assessed here relates to maternal sensitivity.

## Current Study

In this study, we first aimed to gather initial evidence of the applicability of a novel observational instrument (*A&S M-I*; Doodeman et al., 2019; Hoffman et al., 2019) in dyads with infants that were typically developed and infants with a VI. To address our aim, we employed the instrument in two samples of dyads with infants, with and without VI, to assess attunement in the interaction. It was the second aim of this study to investigate whether parental sensitivity influences the extent to which parents’ attune to their infants with and without VI. These findings will provide preliminary evidence to assess and investigate how parent–infant interactions are organized in the presence or the absence of a VI, and inform future clinical work on interventions and parents’ training to adapt to their infants’ needs.

## Study 1: Typically Sighted Sample

### Methods

#### Participants

Twenty-four infants (11 girls, *M*_age_ = 3.31 months; SD_age_ = 0.29) and their mothers (*M*_age_ = 26.12 years; SD_age_ = 5.61) were subsampled from a larger longitudinal study (*N* = 270), the Drakenstein Child Health Study conducted in the Drakenstein sub-district of Paarl, in the Western Cape, South Africa. As per inclusion criteria, infants did not have any impairments. The sample included low SES Black Xhosa-speaking and mixed-race Afrikaans-speaking mother–infant dyads. The study was approved by the Faculty of Health Sciences, Human Research Ethics Committee, University of Cape Town (401/2009), by Stellenbosch University (N12/02/0002), and by the Western Cape Provincial Health Research committee (2011RP45), South Africa.

### Instruments

#### Attunement

**Figure 1:**
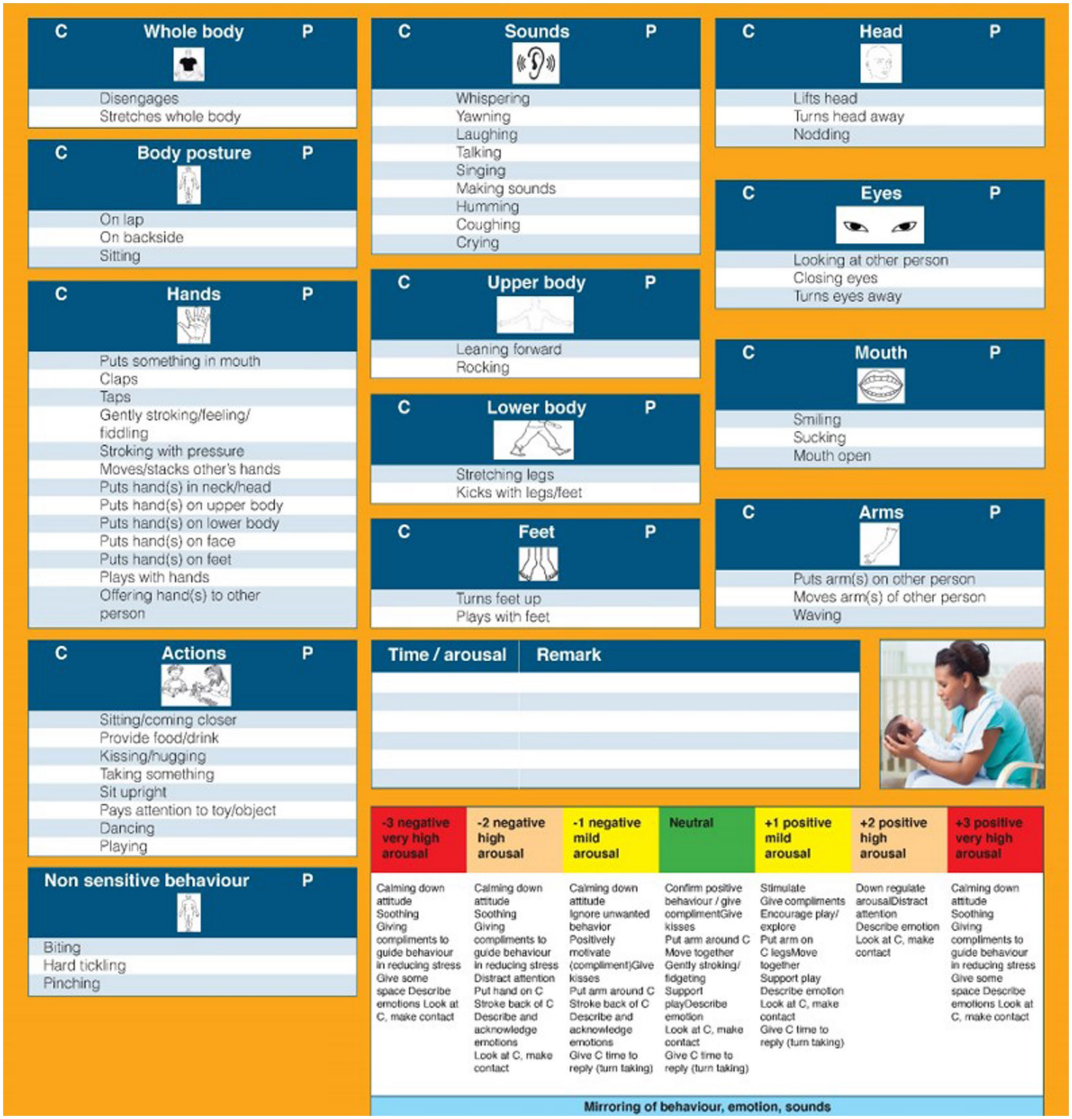
The attune & stimulate mother-infant checklist.

The *A&S M-I* instrument (*A&S-MI*; Doodeman et al., 2019) is an observational instrument, which was used to assess attunement in parent–infant in each of the two samples. Behaviors span across several modalities, from vocalizations, to posture and facial expressions. This is a comprehensive checklist of behaviors that captures different body parts and their motility (i.e., finger movements, arm waving, and foot kicking), and different senses (i.e., audio, tactile, and visual). An illustration of the instrument is provided in Figure 1. The appropriate responding of a parent to the child’s signal (i.e., matching and containing) reflects the ability to attune in the dyad as well as the ability of the parent to stimulate the child to become engaged in the contact or activity (Doodeman et al., 2019). A score of 1 is provided if the behavior is observed, while a 0 score indicates the absence of the behavior. Mothers and infants were video-recorded during a face-to-face interaction of approximately 8 min, and were coded separately, each receiving a score for each behavior in the instrument, for a possible total score of 53. In this study, we aimed to assess whether infants display the behaviors enlisted in the instrument and whether also the parents display the same behaviors. For the first stage of this study, we did not look at time-contingency between infants’ and parents’ behaviors. Next, a correlation coefficient was computed for each dyad between the scores of the infant and the parent. A positive score indexes the presence of attunement, whereas a negative score indicated dis-attunement. The correlation scores can vary between −1 and 1, with higher positive scores suggesting higher dyadic attunement. The scoring was performed by SVS, NH, and PS, and lasted approximately 40 min for each dyad.

#### Parental Sensitivity

*Global rating scale for Mother–Infant Interaction* (GRS; Murray et al., 1996) consists of 44 items assessing the quality of face-to-face mother–infant interaction. Mothers were instructed to play with their infant as they would normally do. Maternal behavior was rated with regard to sensitivity, intrusiveness, remoteness, and depression. Infant behavior was rated on three dimensions, describing the infant’s positive engagement in the interaction, liveliness, and fretfulness behaviors. A final dimension assesses the quality of the overall interaction between mother and infant. For the scope of this study, only maternal sensitivity was included in the analyses. Behaviors were scored on a 5-point Likert scale, with one indicating poor behaviors and five indicating positive behaviors. Maternal intrusiveness, remoteness and depression, and infant fretfulness were reversed coded. The scoring was performed by the fifth author after receiving intensive training in administering and scoring the GRS, achieving an interclass correlations of 0.7–0.9 for all scales which was deemed reliable, as shown in previous reports (e.g., Murray et al., 1996).

## Data Analyses

Attunement of the dyadic interaction was assessed by computing a correlation coefficient of the scores obtained by each partner in the dyad on the instrument, based on whether they displayed the behavior. The average scores were computed on the correlation coefficient of each dyad, as an index of attunement. Next, we assessed whether mother–infant dyads showed behavioral attunement, by performing a one-sample *t*-tests, as an indication of the feasibility of the instrument to capture behaviors in the dyad. Effect sizes are reported using Cohen’s *d*. Lastly, we performed regression analyses between attunement and maternal sensitivity, to identify whether parental sensitivity predicts dyadic attunement.

## Results

### Psychometric Properties of the Attune & Stimulate Mother–Infant Instrument

Two independent observers scored the behaviors, reaching good interrater reliability for infant and maternal behaviors, with an intraclass correlation coefficient (ICC) of 0.785 for infant [CI 95%: 0.570, 0.915] and 0.691 for maternal behaviors [CI 95%: 0.432, 0.869], whereas disagreements were resolved by reaching verbal consensus. Good test–retest reliability with Cohen’s *k* of 0.780 was achieved for infants and 0.739 for mother’s behaviors. Internal consistency analyses revealed acceptable Cronbach alpha coefficient of 0.662 with 0.730 for mothers and 0.546 for infants.

#### Parent–Infant Attunement

Parent–infant dyads reached an attunement mean score of 0.161 (SD = 0.151, Min = −0.120, Max = 0.473), which was significantly above chance [*t*(23) = 15.21, *p* < 0.001, *d* = 1.06].

#### Parental Sensitivity

**Figure 2:**
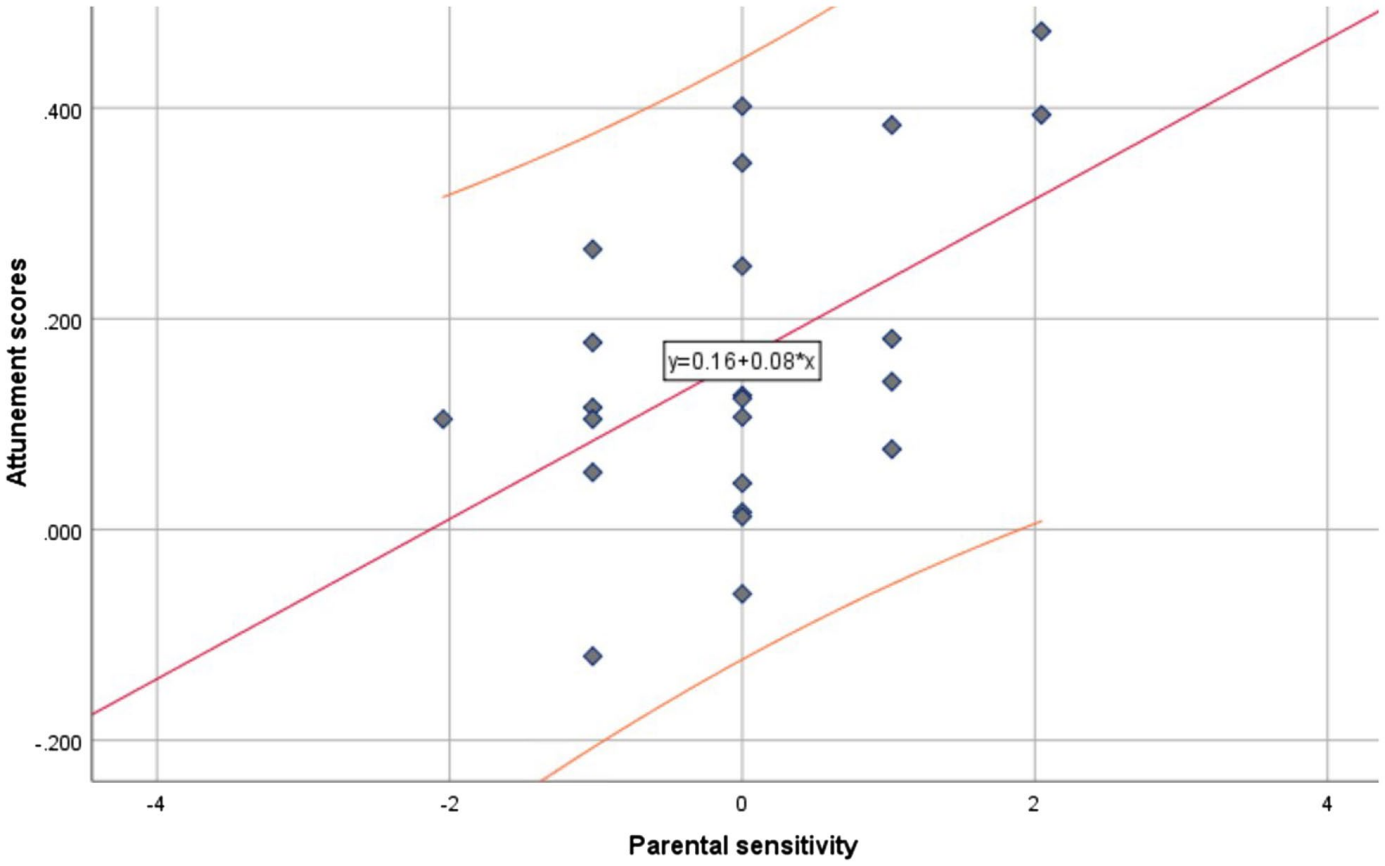
TS sample. Regression slope (red) with confidence interval (orange) of the parental sensitivity scores (*x*-axis) predicting attunement scores (*y*-axis).

Regression analyses revealed that parental sensitivity (*M* = 2.96, SD = 0.845) positively and significantly predicted parent–infant attunement [*β* = 0.49, *t*(23) = 2.70, *p* = 0.013] with 24% of explained variance [*F*(1, 22) = 7.30, *R*^2^ = 0.24, *p* = 0.013; Figure 2].

## Study 2: Visual Impairment Sample

### Methods

#### Participants

Twenty infants with VI and without additional impairments (10 girls, *M*_age_ = 18.90 months; SD_age_ = 3.44) and their parent (*M*_age_ = 30.88 years; SD_age_ = 6.87) were recruited through the collaboration of the Blind Babies Foundation. This is a non-profit organization that provides developmental services for infants with VI, and the patient population in the Infant/Toddler Clinic and the Special Visual Assessment Clinic at the UC Berkeley School of Optometry, both in California, USA. The infants’ VI (i.e., reductions in VA and contrast sensitivity, CS) was assessed by an optometrist at the University of California at Berkeley School of Optometry Infant/Toddler Clinic through a comprehensive visual examination using neurological (visual evoked potential, VEP) and behavioral (preferential looking paradigm, PL) measurements (Dobson, 1994; Norcia et al., 2015). Further information about the VI sample has been reported (Urqueta Alfaro et al., 2018, 2020, 2021). Prior to study procedures, informed consent was obtained from infants’ caregivers. Ethical approval for the original study in which the data was collected was obtained from the University of California, Berkeley Committee for the Protection of Human Subjects (2011-01-2814), USA. Approval to conduct the secondary analysis of the data included in the present study was given by the Radboud University, Nijmegen, Netherlands and Université de Montréal’s Comité d’éthique de la Recherche en Santé (18-116-CERES-D), Canada.

### Instruments

#### Attunement

The *A&S M-I* instrument (*A&S-MI*; Doodeman et al., 2019) was used to score the naturalistic interaction, as in the TS sample. Caregiver–infant interactions were video recorded during naturalistic play for 15 min at their homes. Infant and caregiver could be at any distance from one another, as long as they remained within the viewing range of the video cameras. The toys were placed between child and caregiver. Internal consistency analyses revealed acceptable Cronbach alpha coefficient of 0.728. The scoring for each dyad lasted approximately 40 min in both samples and was performed by SVS, NH, and PS.

#### Parental Sensitivity

The *72-item Maternal behavior Q-sort* (MBQS; Pederson et al., 1990, 1998) was used to assess naturalistic 30-min parent–infant interactions during free play at home. The MBQS describes caregiver behaviors, including the tendency to detect infant’s signals and respond appropriately. The coder gave each item (e.g., “Responds only to frequent, prolonged or intense distress”) a score between 1 (extremely uncharacteristic) and 9 (extremely characteristic) of the caregiver’s behavior. The MBQS provides a “sensitivity criterion” that exemplify a sensitive caregiver. The sensitivity score of a participant parent consists of the correlation between his scores and those of the “sensitivity criterion.” Two research assistants coded each half of the video data. For reliability purposes, six videos were coded by the two coders. Interrater reliability yielded a satisfactory ICC of 0.886 [CI 95%: 0.839, 0.889], in line with previous findings (e.g., Behrens et al., 2014).

## Data Analyses

Attunement of the dyadic interaction was assessed by computing a correlation coefficient of the scores obtained by each partner in the dyad on the instrument, based on whether they displayed the behavior. The average scores were computed on each dyad’s correlation coefficient, as an index of attunement. First, we assessed whether the dyad showed behavioral attunement, by performing 2 one-sample *t*-tests, as an indication of the feasibility of the instrument to capture behaviors in the dyad. Effect sizes are reported using Cohen’s *d*. Lastly, we performed regression analyses between attunement and maternal sensitivity, while statistically controlling for severity of the VI.

## Results

### Psychometric Properties of the Attune & Stimulate Mother–Infant Instrument

Internal consistency analyses revealed acceptable Cronbach alpha coefficient of 0.728.

#### Parent–Infant Attunement

In the VI sample, parent–infant dyads reached an attunement mean score of 0.474 (SD = 0.161, Min = 0.190, Max = 0.758) and this was significantly above chance [*t*(19) = 13.15, *p* < 0.001, *d* = 2.94].

#### Parental Sensitivity

**Figure 3:**
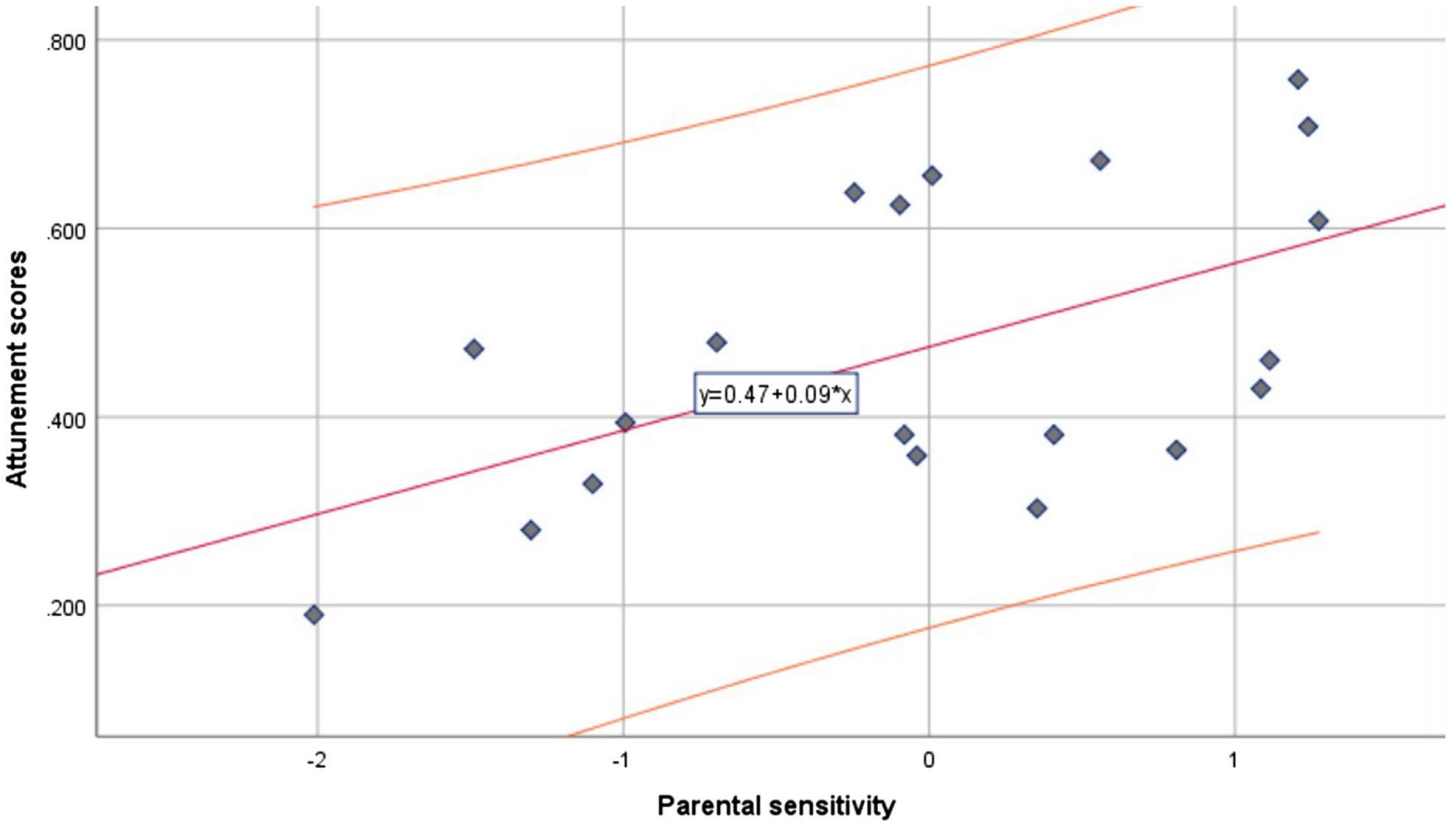
VI sample. Regression slope (red) with confidence interval (orange) of the parental sensitivity scores (*x*-axis) predicting attunement scores (*y*-axis).

Regression analyses revealed a non-significant (all *p* > 0.636; Figure 3) association between sensitivity (*M* = 0.642, SD = 0.092) and attunement, after controlling for VI severity (CS and VA separately, measured both by PL and VEP; Table 1).

**Table 1:** Regression analyses of VI and sensitivity contribution to dyadic attunement.

	***F(df1,df2)***	***R*** ^**2**^	***B***	**SE**	***t***	***p***
Model 1						
	2.84 (2, 17)	0.25				0.086
VA (PL)			−0.05	0.05	−0.95	0.357
VA (VEP)			−0.17	0.25	−0.69	0.502
Model 2						
	0.23 (1, 16)	0.01				0.636
VA (PL)			−0.06	0.06	−1.04	0.315
VA (VEP)			−0.16	0.26	−0.63	0.535
Sensitivity			−0.20	0.42	−0.48	0.636
Model 1						
	0.91	0.09				0.421
CS(PL)			−0.00	0.00	−0.27	0.793
CS (VEP)			−0.01	0.01	−0.69	0.502
Model 2						
	0.62	0.11				0.700
CS (PL)			0.00	0.00	0.07	0.942
CS (VEP)			−0.01	0.02	−0.77	0.450
Sensitivity			0.22	0.56	0.39	0.700

VA, Visual acuity; CS, Contrast sensitivity; PL, Preferential looking paradigm; VEP, Visual evoked potentials; F, *f*-Test value; *df*, Degrees of freedom; *B*, Unstandardized coefficients; SE, Standard error; T, *t*-test value; *p*, Value of *p* with significance level set at 0.05.

## General Discussion

This study aimed to investigate the applicability of a novel observational instrument assessing parent–infant dyadic attunement during naturalistic interactions. We tested the instrument in two separate samples: a typically developed infant sample and a sample of infants with VI. Secondly, we investigated whether parental sensitivity contributes to dyadic attunement.

Our results provide initial evidence for the applicability of the *A&S M-I* observational instrument in parent–infant dyads with an infant with or without a VI. The instrument captures behaviors manifested by the parent and the infant during face-to-face interactions. We found acceptable psychometric properties in both samples, suggesting the applicability of the *A&S M-I* across different settings and populations. In addition, we evidence indicating that this comprehensive instrument captures typical behaviors displayed by infants and their parents, irrespective of the visual capacities. Moreover, in both samples parent–infant attunement scores were positive, indicating that parents and infants overall coordinated their behaviors to one another. Given the study design, this represents correlational evidence and no directionality can be claimed. Moreover, this study provides initial evidence that the instrument captures these behaviors, yet in future work time-contingency of the occurrence of these behaviors should also be tested. It could be the case that although both partners in the dyad manifest the behaviors enlisted in the instrument, the timing may differ depending on infants’ abilities, developmental stage, or maternal sensitivity. Accordingly, future studies should aim at investigating the temporal dependencies (Yale et al., 2003) underlying attunement and pinpoint whether parents follow their infant’s behaviors or, on the contrary, infants follow parents’ behaviors. In a recent study by Vacaru et al. (2022), temporal analyses showed that parents follow their infant’s behaviors in face-to-face interaction possibly reflecting their high engagement in the interaction and affiliation motives.

The second aim of this study was to test the relation between parental sensitivity and attunement in each sample. Our results revealed that sensitivity predicted attunement in the TS sample, explaining up to 25% of the variance, but not in the VI sample. The relation between sensitivity and attunement in the TS sample is in line with prior evidence indicating that parents who are more sensitive show greater attunement (Howe, 2006; Meins, 2013) and contingency (Lohaus et al., 2001a,b; Bigelow et al., 2010) in the interaction with their infant. More surprising, however, is the finding that parental sensitivity does not contribute to attunement between parents and their infants with VI, and as such this warrants further attention. One possible explanation for the lack of a significant relationship between sensitivity and attunement in the VI sample could be that showing attuned behaviors (i.e., moving own arm when the infant moves their arm) may just be part of the normal behavioral repertoire of a parent of an infant with VI, that has likely learn to act with their infant in this (necessary) way. The same instance of attunement in a dyad with a TS infant may be indicative of an interactive element beyond the necessary repertoire. In other words, for parents of an infant with VI, behavioral attunement may be just essential to communicate with their infant, which lacks visual information, but this is not the case for dyads with a TS infant.

Alternatively, it could be the case that parents of infants with TS might engage with their infants mainly through the face and gaze (Colonnesi et al., 2012), but miss some other signals that infants may use, such as kicking or other tactile cues. Parent’s focus around the face region might be greater for infants with TS compared to infants with VI, and indeed, prior research showed that mothers of children with VI tend to be more physical and vocal in the interaction with their child compared to mothers of TS children (Behl et al., 1996). It could be argued that given that infants with VI might not respond with typical gaze and facial expression patterns, parents may attune more to behaviors not centered in the face (e.g., infants’ body movements) which reflect these infant’s preferential communication strategy. Our instrument comprises a spectrum of behaviors related to a large range of body parts and several senses, with the face being just one dimension. Consequently, the TS sample’s lower attunement might reflect TS parents’ face bias. It is also worth mentioning that the VI sample was recruited from clinics where parents of infants with VI were offered training to interact with their infant in a sensitive manner. Although this was not within the scope of our study, these findings in combination with the counseling that the sample was receiving might indicate that parents of infants with a VI have already learned to identify specific signals that their infants use in communication and respond appropriately to these. A complementary explanation lies in the TS sample characteristics. The TS sample might have scored lower on attunement, bearing in mind that this was drawn from a community of low SES population. In contrast, the VI sample included a range of SES levels ranging from low to high incomes. Bearing in mind the intrinsic characteristics in our samples, caution should be exercised in the interpretation of the dyadic attunement difference between the VI and the TS samples.

Moreover, the spread of scores in the scatterplots (Figures 2 and 3) is noteworthy, indicating generally little variance on the sensitivity scores, especially in the TS sample. The low variance is likely due to the small sample sizes, leaving these findings somewhat inconclusive. Further research should aim to replicate these findings with larger samples and multiple assessments of parental sensitivity. Indeed, another possibility of the relation, and lack thereof, between attunement and sensitivity in the two samples separately is the difference in the instruments used to assess parental sensitivity: for the VI sample, the *Maternal Behavior Q-sort* (Pederson et al., 1998) was scored, whereas for the TS sample, the *GRS for Mother–Infant interaction* was used (Murray et al., 1996).

The results of this preliminary study provide important initial evidence on the feasibility and applicability of an observational instrument to assess dyadic attunement in the presence of VI in the infant, comparable to infant with TS, capturing a broad range of behaviors that infants with and without VI manifest and to which parents respond adequately. This study also provides important first evidence of the psychometric properties of the *A&S M-I*, showing acceptable internal consistency, test–retest reliability and predictive validity supported by the relation between sensitivity and attunement. Some limitations worth mentioning are the small sample sizes which should be regarded as preliminary evidence to help larger studies undertake the investigation of the *A&S M-I* in samples with (a)typical development. This may also lay the ground work for future intervention studies to train parents to coordinate their behaviors to those of the infants with or without an impairment. For infants with VIs, it may be particularly useful to educate the parents on the importance of attunement using cues that do not involve gaze. For instance, during the development of the instrument, professionals discussed their observations with the mothers and they provided tips on certain behaviors, such as to mirror the child’s behaviors, provide the child with more time to respond, or also correct insensitive behaviors. By using such a detailed checklist of behaviors it may provide practitioners with an immediate tool for discussion of concrete behaviors that parents can address in order to become more sensitive and attune to their infant’s needs and capacities.

Noteworthy, the *A&S M-I* instrument allows to code for both children and parent behaviors, and in this study, we assessed the absence or the presence of a behavior for each partner separately. While this gives indication of the instrument’s capacity to capture relevant behaviors and to some extent the degree of overlap between mother’s and child’ behaviors, it does not allow to draw conclusions on contingent attunement. This represents a limitation of our study, but an important research venue to undertake in future work. Accordingly, future studies should aim to conduct time-window sequential analyses on mother–child interactions, as earlier proposed by Vacaru et al. (2022) to investigate attuned behaviors.

An additional limitation of this study is the use of different sensitivity questionnaire, which led to different results in the two samples, making it difficult to conclude on the relation between sensitivity and dyadic attunement. In addition, the two samples also differ in the age of the infants and their context (United States vs. South Africa). While this can be noted as a limitation, it can well be seen as a strength of the generalizability of this instrument to capture meaningful interaction behaviors at different ages, in different cultural contexts and beyond visual abilities. Furthermore, future studies should aim at investigating the dimensionality of the instrument in a factor analysis and identify which sensorial modalities may contribute more to the dyadic attunement. Together, this preliminary evidence constitutes a promising venue to pursue in the identification of dyadic interaction between parents and their infants with VI, with central implications for intervention, to prevent malfunctioning parent–infant relationships and negative social developmental outcomes.

## Data Availability Statement

The raw data supporting the conclusions of this article will be made available by the authors, without undue reservation.

## Ethics Statement

The studies involving human participants were reviewed and approved by Faculty of Health Sciences, Human Research Ethics Committee, University of Cape Town (401/2009), by Stellenbosch University (N12/02/0002), and by the Western Cape Provincial Health Research committee (2011RP45), South Africa; University of California, Berkeley Committee for the Protection of Human Subjects (2011-01-2814), United States; Radboud University, Nijmegen, Netherlands; Université de Montréal’s Comité d’éthique de la Recherche en Santé (18-116-CERES-D), Canada. Written informed consent to participate in this study was provided by the participants’ legal guardian/next of kin.

## Author Contributions

VV and AA analyzed the data, interpreted the results and wrote the manuscript. They had equal contribution and share first authorship. AA and NH collected the data. PS and WW provided support with the data analyses and provided input and feedback on the manuscript. MS, HZ, and DS provided input and feedback on the manuscript. All authors contributed to the article and approved the submitted version.

## Conflict of Interest

The authors declare that the research was conducted in the absence of any commercial or financial relationships that could be construed as a potential conflict of interest.

## Publisher’s Note

All claims expressed in this article are solely those of the authors and do not necessarily represent those of their affiliated organizations, or those of the publisher, the editors and the reviewers. Any product that may be evaluated in this article, or claim that may be made by its manufacturer, is not guaranteed or endorsed by the publisher.
