# Corrigendum: Applicability of a novel attunement instrument and its relationship to parental sensitivity in infants with and without visual impairments

**DOI:** 10.3389/fpsyg.2022.967247

**Published:** 2022-07-12

**Authors:** Victorita Stefania Vacaru, Andrea Urqueta Alfaro, Nadia Hoffman, Walter Wittich, Micky Stern, Heather J. Zar, Dan J. Stein, Paula Sophia Sterkenburg

**Affiliations:** ^1^Donders Centre for Cognitive Neuroimaging, Donders Institute for Brain, Cognition and Behaviour, Radboud University, Nijmegen, Netherlands; ^2^Vrije Universiteit, Amsterdam, Netherlands; ^3^Nazareth and Louis-Braille Institute, Integrated Health and Social Services Centres (CISSS), Longueuil, QC, Canada; ^4^School of Optometry, Université de Montréal, Montreal, QC, Canada; ^5^Department of Psychiatry and Mental Health, Faculty of Health Sciences, University of Cape Town, Cape Town, South Africa; ^6^Institut Nazareth et Louis-Braille du CISSS de la Montérégie-Centre, Longueuil, QC, Canada; ^7^MRC Unit on Child and Adolescent Health, Department of Paediatrics and Child Health, University of Cape Town, Cape Town, South Africa; ^8^Department of Paediatrics & Child Health, Red Cross War Memorial Children's Hospital, Cape Town, South Africa; ^9^Risk and Resilience in Mental Disorders Research Unit, South African Medical Research Council, Cape Town, South Africa; ^10^Bartiméus, Zeist, Netherlands

**Keywords:** visual impairment, attunement, parental sensitivity, parent–infant interaction, instrument validation

In the citation of the original article there was a mistake. The second author “Andrea Urqueta Alfaro” was listed as “Alfaro AU.” They should be listed as “Urqueta Alfaro A.”

In the published article, there was also an error in the authorship list, the first two authors share first authorship but the statement of shared first authorship was omitted. The amended authorship list appears below:

Victorita Stefania Vacaru^1,2†^, Andrea Urqueta Alfaro^3,4†^, Nadia Hoffman^5^, Walter Wittich^4,6^, Micky Stern^5^, Heather J. Zar^7,8^, Dan J. Stein^5,9^ and Paula Sophia Sterkenburg^2,10^

^†^These authors share first authorship

In the published article, a number of references by author “Andrea Urqueta Alfaro” were also incorrectly written as “Alfaro, A.U.” but should be “Urqueta Alfaro, A.”

For Urqueta Alfaro et al. (2018) the reference was incorrectly written as “Alfaro, A. U., Morash, V. S., Lei, D., and Orel-Bixler, D. (2018). Joint engagement in infants and its relationship to their visual impairment measurements. *Infant Behav. Dev*. 50, 311–323. doi: 10.1016/j.infbeh.2017.05.010” but should be “Urqueta Alfaro, A., Morash, V. S., Lei, D., and Orel-Bixler, D. (2018). Joint engagement in infants and its relationship to their visual impairment measurements. *Infant Behav. Dev*. 50, 311–323. doi: 10.1016/j.infbeh.2017.05.010”.

For Urqueta Alfaro et al. (2020) the reference was incorrectly written as “Alfaro, A. U., Guthrie, D. M., McGraw, C., and Wittich, W. (2020). Older adults with dual sensory loss in rehabilitation show high functioning and may fare better than those with single sensory loss. *PLoS One* 15:e0237152. doi: 10.1371/journal.pone.0237152” but should be “Urqueta Alfaro, A., Guthrie, D. M., McGraw, C., and Wittich, W. (2020). Older adults with dual sensory loss in rehabilitation show high functioning and may fare better than those with single sensory loss. *PLoS One* 15:e0237152. doi: 10.1371/journal.pone.0237152”.

For Urqueta Alfaro et al. (2021) the reference was incorrectly written as “Alfaro, A. U., Vacaru, S., Wittich, W., and Sterkenburg, P. S. (2021). Identifying when children with visual impairment share attention: a novel protocol and the impact of visual acuity. *Infant Behav. Dev*. 64:101585. doi: 10.1016/j.infbeh.2021.101585” but should be “Urqueta Alfaro, A., Vacaru, S., Wittich, W., and Sterkenburg, P. S. (2021). Identifying when children with visual impairment share attention: a novel protocol and the impact of visual acuity. *Infant Behav. Dev*. 64:101585. doi: 10.1016/j.infbeh.2021.101585”.

In Visual Impairments, Paragraph 1, the citation for “Urqueta Alfaro et al., 2021” was incorrectly written as “Alfaro et al., 2021.” The following amendment has been made:

“Visual impairments during early development may affect the quality of the parent–infant relationship (Howe, 2006; Sterkenburg et al., 2022): infants with VI may not be able to capture the range of their parent's visual cues, whereas TS parents may miss or misinterpret their infant's cues (Nagayoshi et al., 2017; van den Broek et al., 2017). Infants with VI may communicate differently, by using a unique set of signals, such as tactile strategies (Chen and Downing, 2006), which parents may not be aware of and hence may not perceive these as meaningful. A recent study indicated that the lower infant's visual acuity (VA), the lower was their ability to share attention with their parent (Urqueta Alfaro et al., 2021). Consequently, parents may fail to respond to infant's signals and stimulate the infant (Platje et al., 2018) or may become directive and intrusive (for a systematic review see: Grumi et al., 2021). For example, infants who are blind or have a severe VI may not make eye contact with their parents nor engage in reciprocal imitation games of facial expressions, interactions that are documented in TS infants during the first months of life (Beebe et al., 2010; Markova, 2018; Vacaru et al., 2022). Instead, infants with VI may react to their parent's approach by making lips or tongue movements, as well as by waving legs and arms (Preisler, 1991). It is important to note that the impact of VI in child development varies depending on factors such as the severity of the VI (ranging from no light perception to low vision), and the parents' ability to adapt their child-rearing practices to the unique needs of infants with VI (Warren et al., 1997; Lueck, 2008). To alleviate the strain of VI on the parent–infant relation and to mitigate the potential profound detrimental effects on infants' subsequent development, it is crucial to identify the central communicative signals in the parent–infant interaction in the presence of VI. Identifying these signals hold important implications for supporting early intervention programs (Overbeek et al., 2015) to promote parent–infant attunement and psychological wellbeing (Kúld et al., 2020).”

In Study 2: Visual Impairment Sample, Methods, Participants, Paragraph 1 the citation for “(Urqueta Alfaro et al., 2018, 2020, 2021)” was incorrectly written as “Alfaro et al., 2018, 2020, 2021.” The paragraph has been amended as:

“Twenty infants with VI and without additional impairments (10 girls, *M*_age_ = 18.90 months; SD_age_ = 3.44) and their parent (*M*_age_ = 30.88 years; SD_age_ = 6.87) were recruited through the collaboration of the Blind Babies Foundation. This is a non-profit organization that provides developmental services for infants with VI, and the patient population in the Infant/Toddler Clinic and the Special Visual Assessment Clinic at the UC Berkeley School of Optometry, both in California, USA. The infants' VI (i.e., reductions in VA and contrast sensitivity, CS) was assessed by an optometrist at the University of California at Berkeley School of Optometry Infant/Toddler Clinic through a comprehensive visual examination using neurological (visual evoked potential, VEP) and behavioral (preferential looking paradigm, PL) measurements (Dobson, 1994; Norcia et al., 2015). Further information about the VI sample has been reported (Urqueta Alfaro et al., 2018, 2020, 2021). Prior to study procedures, informed consent was obtained from infants' caregivers. Ethical approval for the original study in which the data was collected was obtained from the University of California, Berkeley Committee for the Protection of Human Subjects (2011-01-2814), USA. Approval to conduct the secondary analysis of the data included in the present study was given by the Radboud University, Nijmegen, Netherlands and Université de Montréal's Comité d'éthique de la Recherche en Santé (18-116-CERES-D), Canada.”

The authors apologize for these errors and state that this does not change the scientific conclusions of the article in any way. The original article has been updated.

## Publisher's note

All claims expressed in this article are solely those of the authors and do not necessarily represent those of their affiliated organizations, or those of the publisher, the editors and the reviewers. Any product that may be evaluated in this article, or claim that may be made by its manufacturer, is not guaranteed or endorsed by the publisher.

